# C4b Binding Protein Binds to CD154 Preventing CD40 Mediated Cholangiocyte Apoptosis: A Novel Link between Complement and Epithelial Cell Survival

**DOI:** 10.1371/journal.pone.0000159

**Published:** 2007-01-17

**Authors:** Kevin T. Williams, Steven P. Young, Alison Negus, Lawrence S. Young, David H. Adams, Simon C. Afford

**Affiliations:** 1 The Liver Research Group and MRC Centre for Immune Regulation, Institute of Biomedical Research, The University of Birmingham, Birmingham, United Kingdom; 2 Department of Rheumatology, University of Birmingham Medical School, Birmingham, United Kingdom; 3 Cancer Research UK Institute for Cancer Studies, University of Birmingham Medical School, Birmingham, United Kingdom; Laboratory of Neurogenetics, National Institutes of Health, United States of America

## Abstract

Activation of CD40 on hepatocytes and cholangiocytes is critical for amplifying Fas-mediated apoptosis in the human liver. C4b-Binding Protein (C4BP) has been reported to act as a potential surrogate ligand for CD40, suggesting that it could be involved in modulating liver epithelial cell survival. Using surface plasmon resonance (BiaCore) analysis supported by gel filtration we have shown that C4BP does not bind CD40, but it forms stable high molecular weight complexes with soluble CD40 ligand (sCD154). These C4BP/sCD154 complexes bound efficiently to immobilised CD40, but when applied to cholangiocytes they failed to induce apoptosis or proliferation or to activate NFkB, AP-1 or STAT 3, which are activated by sCD154 alone. Thus C4BP can modulate CD40/sCD154 interactions by presenting a high molecular weight multimeric sCD154/C4BP complex that suppresses critical intracellular signalling pathways, permitting cell survival without inducing proliferation. Immunohistochemistry demonstrated co-localisation and enhanced expression of C4BP and CD40 in human liver cancers. These findings suggest a novel pathway whereby components of the complement system and TNF ligands and receptors might be involved in modulating epithelial cell survival in chronic inflammation and malignant disease.

## Introduction

Regulation of cholangiocyte survival is crucial for maintaining epithelial cell integrity in the biliary tract. Immune-mediated destruction of cholangiocytes in diseases including primary biliary cirrhosis (PBC), allograft rejection and graft versus host disease occurs as a result of increased apoptosis, mediated by activation of tumour necrosis factor receptor family members (TNFr) [1]. We have previously shown that apoptosis of human hepatocytes and cholangiocytes is regulated by co-operative interactions involving CD40 and CD95 (Fas) in which activation of cell surface CD40 by its ligand CD154 results in the induction of Fas ligand (CD178) and autocrine or paracrine activation of Fas [2], [3]. CD40-activated Fas-dependent apoptosis requires sustained activation of the transcription factors cFos/cJun (AP-1) and STAT 3 to overcome transient, short lived NFkB (RelA) activation and tip the balance away from survival towards apoptosis [2], [4]. In contrast CD40 activation of hepatic endothelial cells results in proliferation associated with sustained upregulation of NFkB and an absence of AP-1 activation [5].

The ability of CD40 to mediate epithelial cell apoptosis may have evolved as a mechanism to clear infected cells. In support of this, loss of functional CD154 in patients with X linked hyper IgM syndrome or in mice with targeted deletions of CD154 results in defective clearance of cryptosporidial infections of the biliary tract associated with chronic inflammation, hyperplasia of the biliary epithelium and tumours [6], [7]. Thus CD40 may provide a mechanism to limit cell proliferation and malignant transformation following injury or infectio

Recent studies suggest that C4b binding protein (C4BP), an inhibitor of C3 convertase of the classical and lectin pathways of complement activation [8], [9], can act as a surrogate ligand for CD40 with the potential to activate B cells thereby linking the innate immune system and complement activity [10]. The liver is a major site of synthesis of both soluble and membrane associated C4BP [11] prompting us to investigate whether C4BP could bind CD40 on epithelial cells and thereby modulate cell survival. Herein we report that C4BP can form stable high-molecular weight complexes with soluble CD154 (sCD154), which despite binding CD40, completely inhibit CD40-mediated apoptosis of human cholangiocytes. Furthermore, we show using surface plasmon resonance (Biacore), that C4BP does not bind directly to CD40. Whereas activation of cholangiocyte CD40 by sCD154 produced transient upregulation of NFkB (Rel A), sustained activation of AP-1 (cJun/cFos) and pSTAT 3 resulting in cholangiocyte apoptosis [5], the sCD154/C4BP complex failed to activate these pathways and resulted in inhibition of apoptosis and enhanced cell survival. Thus C4BP can modulate CD40/sCD154 interactions by presenting a high molecular weight multimeric sCD154/C4BP complex that suppresses activation of transcription factors that critically regulate cholangiocyte apoptosis permitting survival without proliferation. These results demonstrate a novel mechanism by which components of the innate immune system can modulate the survival of epithelial cells during inflammation and infection. The results may also have important implications for cellular transformation during epithelial malignancy.

## Results

### C4BP prevented CD40 mediated cholangiocyte apoptosis but did not cause cell proliferation

**Figure 1:**
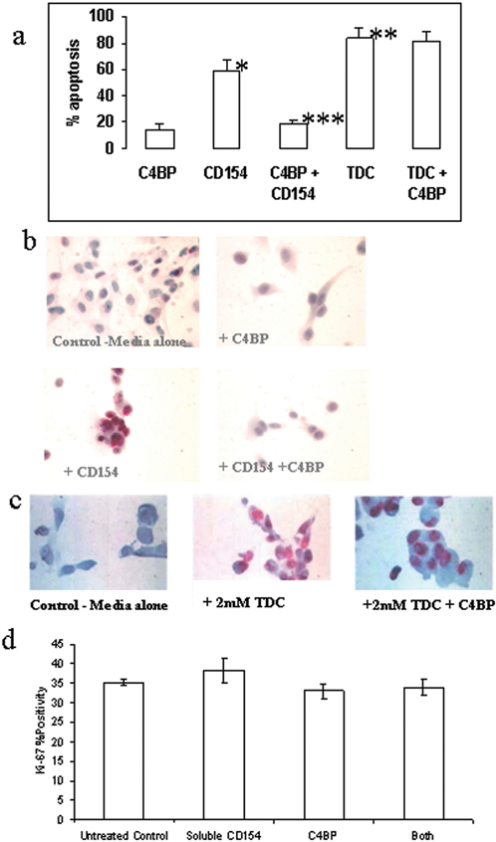
Panel a) This histogram shows inhibition of sCD154 mediated apoptosis by C4BP but not apoptosis induced by 0.2 mM TDC. * sCD154 or **TDC induced similar levels of cholangiocyte apoptosis when experimental error was taken into account (59.7%+/−7.5 and 83.4%+/−7.7 and respectively) relative to control (p<.005)***. C4BP + sCD154 reduced apoptosis to control levels (p<0.005) whereas C4BP had no effect on TDC induced apoptosis (81.5%+/−6.7). C4BP/sCD154 also had no effect on TDC mediated apoptosis (data not shown) Panel b and c show representative cytospins stained for fragmented DNA using ISEL. Panel d) Histogram summary of the effects of sCD154 and C4BP on Cholangiocyte proliferation. Primary human cholangiocytes were cultured in 24-well culture plates and simulated with either sCD154, C4BP or a mixture of both. Following incubation for 24 hours, the cells were fixed and proliferation assessed by immunohistochemical staining for Ki-67 antigen. No significant difference was seen between the un-stimulated controls and treated samples, implying that sCD154, C4BP or the mixture had any effect on cholangiocyte proliferation. These data represent the mean of three different counted areas per well repeated for three different liver preparations.

On its own C4BP at concentrations of up to 50 ug/ml had no effect on cholangiocyte apoptosis whereas treatment with 1 ug/ml sCD154 increased apoptosis from 14.8+/−3.8% ISEL positive cells to 59.1+/−7.5% (p<0.005) at 24 hours. When cells were treated with a combination of sCD154 and C4BP the induction of apoptosis observed with sCD154 alone was completely inhibited at C4BP concentrations of 5 ug/ml or greater (18.2+/−3.4% ISEL positive cells). In contrast, addition of C4BP to the bile acid Taurodeoxycholic acid (TDC) had no discernable effect (TDC induced apoptosis 83.4+/−7.63% cells; TDC and C4BP treated cells 81.5+/−6.7% ISEL positive cells) (figure 1a–c). The addition of C4BP/sCD154 also had no effect on TDC mediated apoptosis (data not shown).

Ki67 nuclear staining demonstrated that the sCD154/C4BP complex did not effect cholangiocyte proliferation compared with untreated control cells (figure 1d)

### Analysis of C4BP/CD40/CD154 interactions with surface plasmon resonance

**Figure 2:**
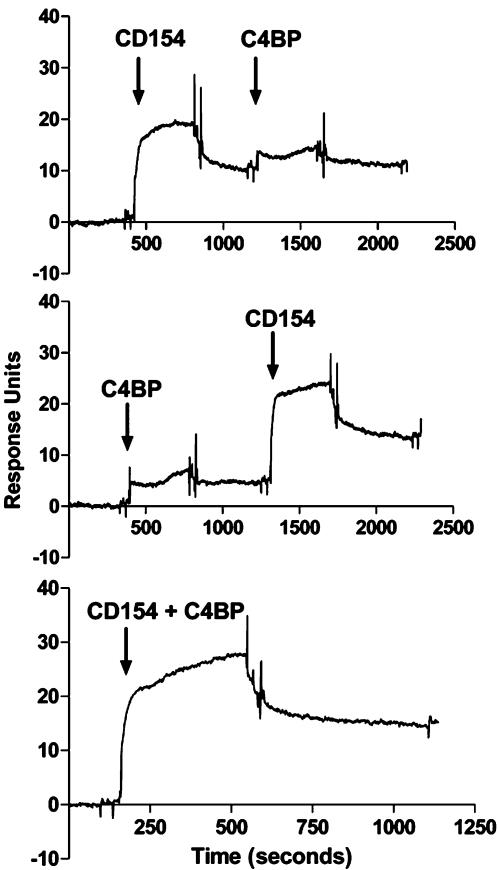
Surface Plasmon Resonance (Biacore) analysis – This figure shows the sensogram for interactions between immobilised CD40, sCD154 (10 ug/ml) and C4BP (40 ug/ml). The human CD40 fusion protein was immobilised onto an activated CM5 chip and sCD154, C4BP or a mixture of both was flowed across the surface of the chip in various combinations allowing adequate time for both association and dissociation events. The chip was regenerated using between experiments using 2 M glycine. Any change in surface plasmon resonance due to binding of the soluble proteins to the immobilised CD40 was seen as a change in response units on the sensogram.

Biacore analysis revealed the predicted binding between immobilised CD40 and sCD154. However no binding was observed between CD40 and C4BP under any conditions. The possibility that sCD154 occupancy obscured potential C4BP-binding sites on CD40 was excluded by challenging immobilised CD40 with C4BP first, followed by sCD154. In this experiment binding and dissociation of the ligand could be seen, whereas no interaction between C4BP and CD40 was observed (figure 2).

**Figure 3:**
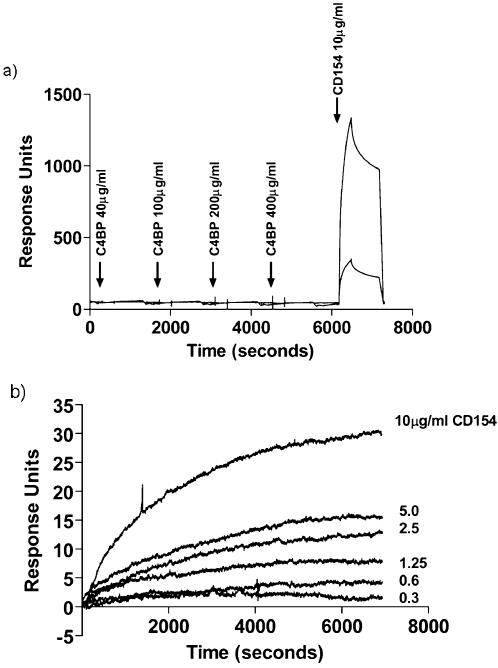
Biacore analysis – Panel a) This figure shows the sensogram trace for the titration of C4BP binding across immobilised CD40. CD40 fusion protein was immobilised on an activated CM5 chip, to yield 2000, 500 and 100 response units on three separate channels. Soluble C4BP was then flowed across the surface of the chip at increasing concentrations. The chip was regenerated between the different concentrations using 2M glycine. No binding or dose-response was seen. The integrity of the CD40 was confirmed by the determining the ability of the receptor to subsequently bind sCD154. Panel b) This figure shows the sensogram for sCD154 binding to immobilised C4BP. C4BP was immobilised on an activated CM5 research grade chip. sCD154 was then flowed across the chip at increasing concentrations up to 10 ug/ml. The chip was regenerated between concentrations using 2 M glycine. Dose dependent binding of sCD154 was observed across the concentration range. No binding was observed with an irrelevant fusion protein (data not shown).

To determine whether the lack of CD40/C4BP interaction was due to the concentration of C4BP used in the system, a range of C4BP concentrations were tested from 40 µg/ml up to 400 µg/ml (figure 3a). No interaction was seen between CD40 and C4BP, even at the highest achievable protein concentration. The possibility that immobilisation of CD40 had altered the structural conformation of the protein thereby preventing binding to C4BP was eliminated by demonstrating that CD40 was able to bind sCD154 before and after titration of C4BP (figure 3a). Subsequent dissociation of the sCD154/C4BP complex was also clearly seen over time.

To confirm the findings and to determine whether C4BP could bind any component of the CD40/CD154 dyad, experiments were carried out in which C4BP was immobilised on a CM5 chip and soluble CD40 (sCD40) or sCD154 added. C4BP was again unable to bind sCD40, confirming the findings with immobilised CD40. However, C4BP did bind sCD154 in a dose-dependent manner at concentrations of between 0.313 µg/ml to 10 µg/ml (figure 3b). Under similar conditions, C4BP did not bind to either immobilised CD95 (Fas) or CD178 (Fas Ligand) and did not affect the ability of immobilised CD95 to bind soluble CD178 (Data not shown).

### Detection of high molecular mass C4BP/sCD154 complexes by gel filtration

**Figure 4:**
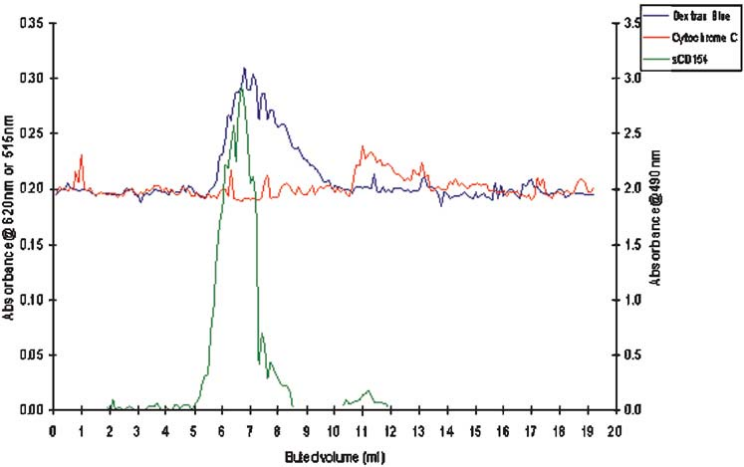
Fractionation of C4BP/sCD154 complex by gel filtration. C4BP and sCD154 were incubated together at 37°C for 1 hour. After this time the solution was eluted on a Sepacryl-300 with 100 ul fractions collected up to a final eluted volume of 20 ml, which encompassed void volume through to the lower limit of the fractionation range ( Dextran blue to cytochrome C). Fractions were assayed for the presence of sCD154 using a commercially available ELISA kit.

Further confirmation of the binding interaction demonstrated by Biacore was obtained by gel filtration chromatography using Sephacryl - 300 where the presence of high molecular weight C4BP/sCD154 complexes was detected. One hundred ul fractions were collected from the column and assayed for the presence of sCD154 using a commercially available ELISA kit. Column void volume was determined using dextran blue exclusion. Cytochrome C was used to identify the maximum elution volume for proteins in excess of 14kDa. High molecular mass sCD154 was detected in fractions that corresponded almost exactly to the elution volume for Dextran blue, confirming the presence of C4BP/sCD154 complexes in excess of 1500kDa (figure 4). The small trailing edge of the major pear (between 7–9 ml elution volume) suggests that a small proportion of intermediate sized complexes of C4BP/sCD154 may also be present but were not clearly separable using this methodology. sCD154 was also detected in fractions corresponding to cytochrome C, demonstrating that some unbound, sCD154 was also present, representing either an excess of sCD154 or a small fraction that had dissociated from the C4BP/sCD154 complex during the experiment.

### NFkB (Rel A) cJun/cFos and STAT 3 activation in cholangiocytes following co-incubation with CD154/C4BP

**Figure 5:**
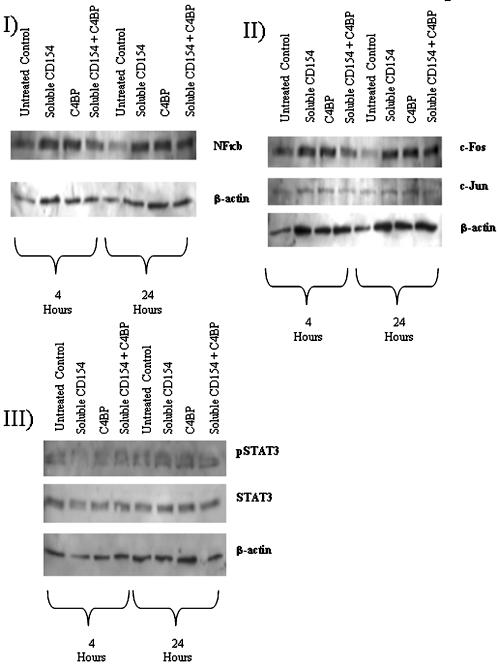
Representative Western blots showing NFkB, c-Fos and c-Jun and pSTAT 3 levels in response to CD154/C4BP stimulation. Aliquots of nuclear or cytoplasmic extracts as appropriate (40 ug protein) from cultured and stimulated primary human cholangiocytes were probed for NFkB (panel I); c-Fos and c-Jun(panel II) or pSTAT3 content. Blots were stripped and re-probed for beta-actin which allowed for normalisation of data for variations in protein loading.

**Figure 6:**
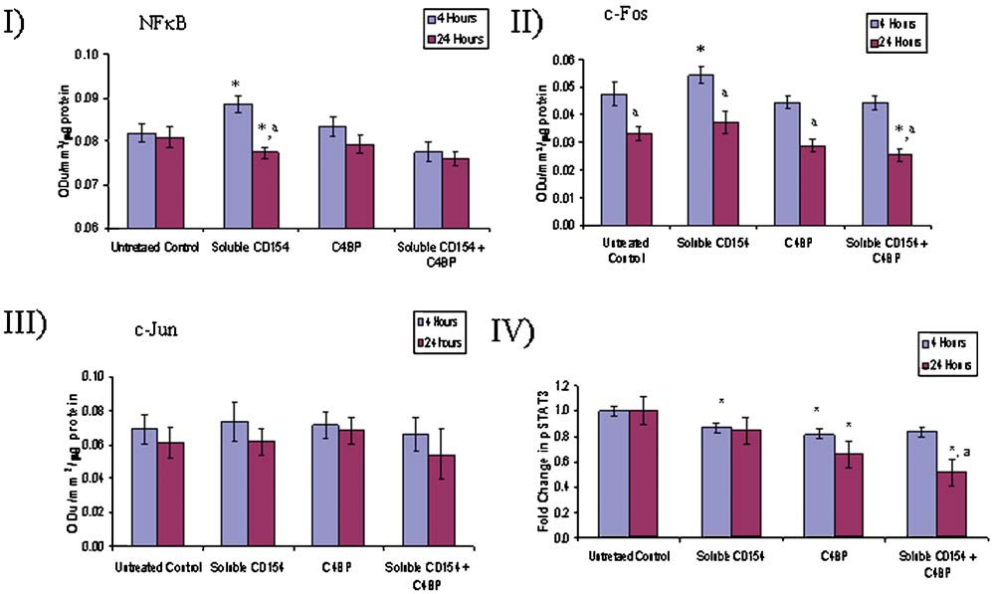
Densitometry Histograms showing changes in NFkB, c-Fos and c-Jun and pSTAT3 levels in response to sCD154 and C4BP stimulation determined using Western blotting (see figure 5). Changes in levels were assessed by densitometric analysis. These data show that the CD154/C4BP complex had no significant influence upon levels of NFkB or c-Jun, but did decrease levels of c-Fos at 24 hours compared with either unstimulated 24 hour control or the stimulated level observed at 4 hours. *p<0.05 a p<0.05These data show that stimulation of cells with CD154/C4BP complex resulted in a lower level of pSTAT3 which was sustained at 24 hours. *p<0.05 c.f. time point matched untreated control a p<0.05 c.f. treatment matched 4 hour time point.

We have previously shown [3], [5] and confirmed here, that activation of CD40 on cholangiocytes results in a transient rise in NFkB activation and a sustained activation of ERK/JNK and cFos/cJun (AP-1 subunits) and STAT 3 phosphorylation associated with death by apoptosis [5]. Activation of cholangiocyte CD40 by sCD154 in the presence of C4BP resulted in no statistically significant increase in transient nuclear NFkB levels compared to control cells (media alone) when data from all four separate experiments was analysed using laser densitometry. However, in contrast to sCD154 alone, C4BP/sCD154 complexes induced short-lived upregulation of the cFos subunit of the AP-1 heterodimer without an increase in cJun and pSTAT3 was downregulated (figure 5 and 6).

### Immunolocalisation of C4BP and CD40 in human liver tissue

**Figure 7:**
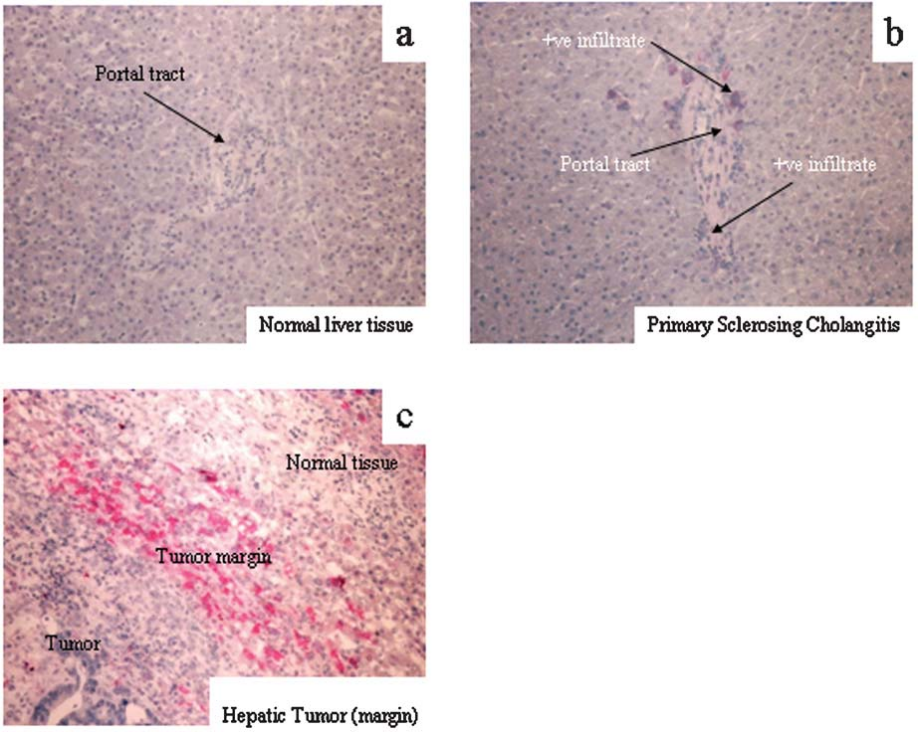
Immunolocalisation of C4BP in human liver tissue. This figure shows three representative sections of liver tissue stained for C4BP. Panel a shows normal liver tissue which is predominantly negative. Panel b shows PSC liver tissue showing the presence of strongly staining inflammatory cells surrounding the portal tract and within the sinusoids. Panel c shows hepatic tumour tissue taken from a hepatic resection for secondary liver cancer (colorectal hepatic metastasis) where very strong staining was observed in the tumour tissue and the inflammatory infiltrate at the tumour margin.

Little C4BP or CD40 expression was detected in normal liver tissue; occasional portal vessels stained weakly for C4BP (figure 7 panel a) and weak CD40 staining was detected on perivenular hepatocytes, sinusoidal endothelial cells and leukocytes infiltrating portal tracts. Similar staining patterns were seen in liver biopsies from patients with end-stage alcoholic liver disease. In PBC weak staining for CD40 was detected within the inflammatory infiltrate, sinusoidal endothelium and on proliferating ductules with C4BP detected in occasional periportal hepatocytes and within the inflammatory infiltrate. Stronger staining was seen in liver tissue from patients with primary sclerosing cholangitis (PSC) where the inflammatory infiltrate, sinusoidal endothelium and ductules stained positively for CD40 and periportal hepatocytes and inflammatory cells for C4BP (figure 7 panel b).

The staining pattern was different in liver tumours. Strong staining for CD40 and C4BP was detected in tumour cells in hepatocellular carcinoma and the tumour stroma, inflammatory cell infiltrate and vessels were also positive. In cholangiocarcinoma a similar pattern of expression was detected The strongest staining for both C4BP (figure 7 panel c) and CD40 was observed in the peri-tumour stroma at the margins of hepatic metastases from colorectal carcinoma where tumour cells, stromal cells, the surrounding inflammatory infiltrate and necrotic centres stained strongly for C4BP. CD40 expression also showed a similar pattern of staining but was absent from the necrotic centres. Hepatocytes in the surrounding normal tissue were predominantly negative for C4BP and CD40 (figure 7 panel c).

**Figure 8:**
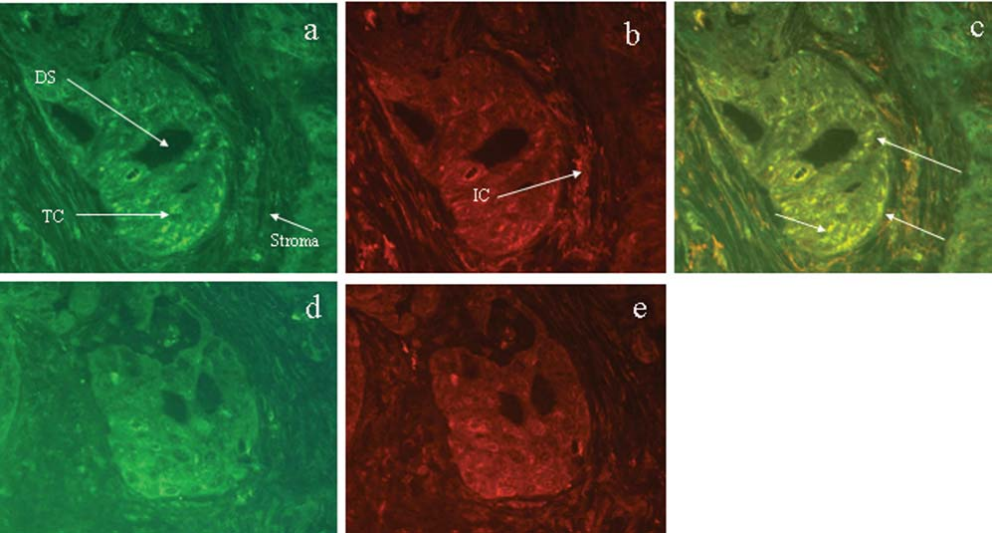
Co-localisation of C4BP and CD40 in liver tumour tissue using dual immunofluorescence. Panels a–c shows a representative section of tumour tissue from a patient with cholangiocarcinoma stained for C4BP (green - FITC) and CD40 (red - PE ). In panel a, the arrows identify an epithelial ductular structure (DS) surrounded by tumour cells (TC) and stromal tissue. Positive C4BP staining is seen within the epithelia, tumour cells, and in mononuclear infiltrate in the surrounding stromal tissue. Panel b shows the same tissue section stained for CD40 with the arrow identifying the inflammatory cells within the surrounding stroma. Panels d and e shows a sequential section from the same specimen where the primary antibodies have been substituted for non immune serum (control). Panel c shows the merged image for panels a and b. The bright yellow areas indicate regions of C4BP and CD40 co-localisation within the epithelial cells of the ductular structure, many surrounding tumour cells, and the inflammatory cells within the surrounding stromal tissue.

In selected samples of tumour tissue we carried out dual immunofluorescence to look for cellular co-localisation of CD40 and C4BP. Figure 8 shows a representative section from a cholangiocarcinoma specimen. C4BP and CD40 co-localised to ductular structures within the tumour as well as surrounding tumour cells. Cells within the surrounding stroma including the mononuclear infiltrate also stained positively for both proteins.

## Discussion

Whilst constitutive expression of CD40 is low in the liver during normal circumstances, it is widespread and increased during inflammatory liver disease implying that it has a pivotal role in regulation of host responses to injury [2], [3], [5], [12]. Expression of CD154, the natural ligand for CD40 is confined to a subset of tissue-infiltrating T cells, macrophages and platelets and is tightly regulated [3], [13], [14], [15]. On T cells expression of CD154 is transient requiring the constant presence of cytokines such as IFNγ and contact dependent mechanisms to permit mRNA stabilisation and sustained expression of the protein [16]. These observations suggest that CD40/CD154 interactions in vivo are likely to be regulated by factors that influence expression and function of the ligand, CD154 as well as the receptor CD40 [17].

The consequences of CD40 ligation vary depending on cell type. In B cells CD40 ligation results in cell survival, immunoglobulin isotype class switching and upregulation of ICAM-1 and CD86, functions which are critical in determining B cell fate and function [18]. On fibroblasts CD40 ligation results in upregulation of ICAM 1, VCAM-1, IL-6, and proliferation [19]. On endothelial cells, CD40 ligation results in NFkB-dependent cell proliferation [4] and upregulation of adhesion molecules and chemokines [20], suggesting a role for CD40 in leukocyte recruitment during inflammation. CD40/CD154 interactions can also regulate epithelial cell fate [2], [3], [4], [5], [21], [22]. The outcome of CD40 ligation will depend on several factors including ligand concentration, mode of presentation, receptor density, and the activation of NFkB-dependent survival factors [23], [24]. Such complex mechanisms possibly explain the diverse consequences of CD40 activation on epithelial cells include activation, turnover and malignant transformation [25], [26].

Until recently it was believed that CD40 interacted with one partner only, CD154. The report in 2003 by Brodeur and colleagues suggested that the structurally unrelated complement inhibitor C4BP could also bind CD40 and function as a surrogate or competing ligand in place of CD154. These observations suggested a novel interface between complement and B cell activation. C4BP is synthesized by hepatocytes and activated monocytes [27], [28] and upregulated by inflammatory cytokines including IFN-γ, IL-6, and TNF-α [28], [29] and by glucocorticoids [30]. In addition to binding C4b, C4BP also has binding sites for protein S [11], heparin [31], bacterial proteins [32] and serum amyloid protein [33]. Because the liver is a major site of C4BP synthesis, we investigated whether C4BP could modulate CD40-mediated responses in cholangiocytes. We show that at concentrations which are likely to be physiologically relevant [9], [11], soluble C4BP completely abrogates cholangiocyte apoptosis induced by sCD154 confirming that C4BP can alter the outcome of CD40 signalling on epithelial cells. This effect was specific for CD40/sCD154 because C4BP had no effect on apoptosis induced by the cytotoxic bile acid TDC (figures 1a–1c). Assessment of Ki67 nuclear proliferation antigen showed that the presence of C4BP maintained cell survival without causing proliferation, inferring that cholangiocytes incubated with sCD154 together with C4BP were held in G^0^ since Ki67 detects all cells in cycle (figure 1d).

Given the report by Brodeur et al [10] we thought that the most likely interpretation was that C4BP had bound cholangiocyte CD40 directly and either inhibited or displaced CD154 binding thereby modulating CD154-mediated signals. However, when we investigated the nature of the C4BP/CD40/sCD154 binding interaction using surface plasmon resonance (Biacore) analysis with purified proteins, we found that CD40 was unable to bind C4BP despite retaining the ability to bind sCD154 (figure 2). An alternative explanation was that sCD154 obscured putative C4BP binding sites or that C4BP could not bind CD40 under these specific conditions. This explanation can be excluded because when we repeated the experiments with C4BP followed by sCD154 and found that binding of C4BP to CD40 was still negligible although sCD154 subsequently bound as expected (figure 2 middle panel). These data confirm that on its own C4BP is unable to bind immobilised CD40. When a mixture of sCD154 and C4BP was flowed over the chip (figure 2 lower panel) binding was achieved confirming in agreement with Brodeur and collegues, that the presence of C4BP did not prevent sCD154 binding to CD40 (see below). Altering the concentration of C4BP by 10 fold, changing the coating concentration of immobilised CD40 or flowing sCD40 over immobilised C4BP had no effect, confirming that C4BP is incapable of binding CD40 under any of the conditions described here.

In theory it remains possible that the recombinant CD40 used in our experiments may differ in tertiary structure to its native form making it incapable of binding C4BP. We feel this is unlikely however because the recombinant protein retains full functional ligand binding activity either when immobilised directly on the BiaCore chip or coupled via immobilised monoclonal antibody specific for CD40 (data not shown).

Thus we remain uncertain why some of our data differ from those published by Broduer and collegues [10]. The presence of CD154 either on a subset of B cells or in a non B cell subpopulation of peripheral blood mononuclear cells could be a factor in their studies. The absence of a C4BP effect in cells from patients with CD40 mutations is more difficult to reconcile with our data, although it is possible that the mutation caused a more generalised defect rendering cells unresponsive to TNFr mediated stimuli. Our data provide an explanation for some of the effects they reported on restoration of class switching and proliferative responses in cells with CD154 mutations. If C4BP binds the mutant CD154 protein it could promote more effective crosslinking of CD154 and CD40 and thereby restore binding and functionality.

Our data confirm that C4BP does not prevent the binding of sCD154, or in our hands, the C4BP/sCD154 complex to CD40. Biacore analysis showed dose-dependent saturable binding of C4BP to increasing concentrations of sCD154 (figure 3b). Although the Biacore data are convincing in themselves, we thought it important to confirm the formation of C4BP/sCD154 complexes using an alternative method, gel filtration, which provided further evidence that CD154 forms stable high molecular weight complexes with C4BP (figure 4). We then went on to investigate the effect of C4BP/sCD154 complex on the major signalling pathways involved in primary cholangiocyte survival, NFkB (RelA), AP-1 (cJun/cFos) and STAT 3. We have previously shown that activation of cholangiocyte CD40 leads to transient RelA expression and sustained cJun/cFos and STAT 3 expression [3], [4] resulting in apoptosis. Surprisingly, despite the ability to bind CD40 when the C4BP/sCD154 complex was used to activate CD40 on cholangiocytes, we were unable to detect transient activation of NFkB or sustained STAT3 phosphorylation and saw only transient activation of the cFos subunit of the AP-1 heterodimer (figure 5 and 6). This provides a possible reason why the presence of C4BP can prevent sCD154 mediated cholangiocyte apoptosis and suggests further complexities to CD40 mediated signalling in relation to cholangiocyte survival.

Our previous comparisons of CD40 mediated NFkB and AP-1 signalling in cholangiocytes and endothelial cells provide evidence for cell-specific differences in transcription factor activation following CD40 ligation [4], [5]. In contrast to cholangiocytes, activation of CD40 on endothelial cells resulted in sustained activation of NFkB and no effect on the AP-1 pathway, the net result being cell proliferation. In the present study, our initial predictions were that the C4BP/sCD154 complex would function to crosslink CD40 more efficiently than sCD154 alone leading to more effective receptor crosslinking and as a consequence, an increase in NFkB and AP-1 (cJun/cFos) activation in cholangiocytes. However, this was not the case and the C4BP/sCD154 complex suppressed the activation of these signalling pathways resulting in cell survival in the absence of proliferation.

The ability of C4BP to modulate the outcome of sCD154 interactions with CD40 suggests the complement system may be involved in regulate epithelial cell survival in the liver. Prolongation of epithelial cell survival is important to facilitate normal protective immune responses, but if sustained could theoretically facilitate malignant transformation. Furthermore, C4BP could potentially modulate CD40 signalling in other cells including endothelial cells, infiltrating leukocytes and stromal cells, which could lead to perpetuation of inflammation. A link between inflammation and malignancy is supported by our finding that C4BP is expressed strongly in the reactive stroma at tumour margins in association with CD40 expression on tumour cells (figure 7 & 8). Interestingly, C4BP protein was also strongly expressed in inflammatory cells and proliferating bile ductules in PSC, a disease associated with a high risk of for malignant transformation of biliary epithelium and the development of cholangiocarcinoma. Thus the presence of C4BP in close proximity to tumor cells expressing the CD40 receptor could suppress the induction of CD40-mediated apoptosis by CD154 effector cells and favour tumour cell survival. The presence of C4BP in this setting may also prevent complement mediated lysis of the tumour cells.

## Materials and Methods

### Isolation and culture of primary cholangiocytes

Cholangiocytes were isolated from a) liver tissue obtained from fully informed consenting patients undergoing transplant surgery for end-stage liver disease b) normal donor liver surplus to transplant requirements. Ethical permission for the use of human liver tissue for research was granted by the local research ethics committee (ref 06/Q702/61)

The cells were isolated and cultured according to the protocol previously described [3] with the minor modification of substitution of 10% v/v heat inactivated foetal calf serum for 10% v/v heat inactivated human serum (HD Supplies UK) in the culture medium. Primary isolated cells were expanded in 75 ml tissue culture flasks for a maximum of 7 passages to ensure phenotypic stability. Prior to carrying out the experiments, cells were recovered via gentle trypsinization and plated onto collagen coated 4 well chamber slides (supplier) for 24–48 hours at an initial density of 2×10^5^ cells per well in 0.5 ml of culture medium.

### The effect of C4BP on CD154/CD40 mediated cholangiocyte apoptosis and proliferation

The cholangiocyte cultures were incubated for up to 24 hours in a) medium alone b) 1 ug/ml recombinant sCD154 (Alexis Biochemicals product no. ALX-522-015), a concentration previously shown to provide optimal induction of apoptosis in these cells [3] c) 1 ug/ml CD154+0.5–50 ug/ml purified native C4BP [11]. In order to exclude a direct effect of C4BP were incubated with d) 0.5–50 ug/ml C4BP alone e) C4BP in the presence of 0.2 mM of the proapoptotic bile acid TDC [12]. At the end of the experiments, the cells were fixed in methanol and assessed for apoptosis using DNA in situ end labelling [3], [5], [15]. Data from the initial experiments informed us that 5 ug/ml C4BP was the minimum concentration required to completely inhibit apoptosis induced by 1 ug/ml sCD154. All subsequent experiments were therefore carried out using these concentrations unless stated otherwise. Cellular proliferation was assessed by measuring Ki-67 after epitope retrieval. Briefly, cells were fixed in situ with methanol and treated with cells with W-CAP Tec buffer pH 8.0 (Bio-Optica, Milan, Italy) for 40 minutes at 96°C. Plates were washed twice at room temperature with Tris-Buffered Saline (TBS, pH 7.6) containing 0.1% Tween-20 (Sigma, Dorset, UK) and cells stained with mouse monoclonal anti-human Ki-67 (Dako Ltd, Cambridge, UK) at a 1/25 dilution at room temperature for 1 hour and washed twice as above. Bound Ki-67 monoclonal antibody was then visualised using a ChemMate DAKO Envision Detection Peroxidase/DAB Kit (Dako Ltd, Cambridge, UK; according to the manufacturer's instructions), followed by counter-staining with Meyer's Haematoxylin (VWR International Ltd, Dorset, UK) for 20 seconds. Cells were scored for the presence of Ki-67 positive nuclei using light microscopy, counting three different random areas per well (min 200 cells/well).The experiment was carried out using cells obtained from three different liver preparations.

### Analysis of C4BP/CD40/sCD154 binding interactions by surface plasmon resonance (Biacore)

C4BP/CD40/sCD154 binding interactions were characterised by Surface Plasmon Resonance(SPR) using the Biacore 3000 system (Biacore AB) fitted with CM5 research grade chips [14]. Experiments were carried out at 25°C using Biacore HBS-EP buffer (10 mM HEPES pH 7.4, 150 mM NaCl, 0.005% surfactant P20). CM5 chips were activated by a 7-minute injection (5 µl/min) of a solution containing a 1∶1 mixture of 0.2 M *N*-ethyl-*N*9-(dimethylaminopropyl) carbodiimide (Sigma) and 0.05 M *N*-hydroxy-succinimide (Sigma). One channel was blocked using a 5-minute injection (10 µl/min) of 1 M ethanolamine (Sigma), in order to serve as the control/reference channel.

In the initial experiments, a human CD40 murine-IgG fusion protein (Ancell) was immobilised on channels 2–4 of the chip, at a concentration of 10 µg/ml and a flow rate of 5 µl/min for 10 minutes. After immobilization, each surface was blocked by a 5-minute injection of 1 M ethanolamine at pH 8.5 at a rate of 10 µl/min. Following immobilization and blocking, sCD154 (the analyte) was passed over the surfaces for 5 minutes at a concentration of 10 µg/ml in HBS-EP buffer, followed by 5-minute dissociation. After this time, purified C4BP [11] was passed over the chip surfaces for 5 minutes at a concentration of 40 µg/ml, followed by 5-minute dissociation. Chip surfaces were then regenerated by two 1-min injections of 2 M glycine (pH 2.0, Sigma).

Once regenerated, the experiment was repeated flowing C4BP (40 ug/ml) across the chip first followed by sCD154 (10 ug/ml) in HBS –EP buffer, for 5 minutes on each occasion, followed a 5-minute dissociation interval. After regeneration as described above, a mixture of C4BP and sCD154 was flowed across the channels on the chip for the same lengths of time and dissociation as described. Any protein–protein interactions between sCD154 and/or C4BP and the immobilized CD40 were reported as sensograms with data recorded as response units (ru) versus time (seconds).

In subsequent experiments, recombinant CD40 (10 µg/ml) was immobilised on an activated CM5 chip at 2000, 500 and 100 ru on channels 2, 3 and 4 respectively. After immobilization, each surface was blocked as described, and C4BP, sCD154 or a mixture of both passed over the surfaces. Chip regeneration was achieved by 2×1 min injections of 2 M glycine between each experiment. A titration was then set up using increasing concentrations of C4BP spanning a range from 40 µg/ml to 400 µg/ml. Each concentration was flowed across the surface of the chip (5 µl/min) for 5 minutes, followed by 5 minutes dissociation. Chip surfaces were regenerated as described before and the subsequent concentration was examined. Protein–protein interactions were recorded as sensorgrams as described above.

In view of the failure to demonstrate interaction between CD40 and C4BP (see results), we carried out an additional experiment where C4BP was immobilised on the chip surface. The chip was activated and one channel blocked as described to serve as the reference/control. C4BP (20 µg/ml) was immobilised onto channels 2, 3 and 4 at pH values pf 4.2, 5.0 and 5.6 respectively (to confirm optimal pH for immobilisation). An injection rate of 5 ul/min for 10 minutes was used. Each surface was subsequently blocked with 1×5-minute injection of 1 M ethanolamine at pH 8.5 at a rate of 10 µl/min. sCD154 was then passed over the chip at concentrations from 0.31 µg/ml to 10 µg/ml. Each concentration was flowed across the surface of the chip at 2 µl/min for 120 minutes, followed by 60 minutes dissociation. Surfaces were regenerated as described before and the subsequent concentration was examined and protein–protein interactions between C4BP and sCD154 were reported as sensorgrams. In additional control experiments the ability of C4BP to bind immobilised CD95 (Ancell, Bayport, USA, cat no. 506–020) or CD178 (Alexis Biochemicals, UK, cat no. ALX-522-001) was assessed under the same conditions.

### Analysis of C4BP/sCD154 interaction by gel filtration

A mixture of C4BP and sCD154 (5 ug∶1 ug) was incubated at 37°C for 1 hour with occasional agitation prior to gel filtration chromatography on Sephacryl–300 (separation range (10kDa–2500kDa) under the following conditions. Sephacryl-300 (Pharmacia) was poured into a 20×1 cm glass gel filtration column (BioRad) and packed using gel filtration buffer (0.1 M NaPO_4_, pH 7.4, containing 0.15 M NaCl) at a flow rate of 1 ml/min. The column was then washed with the equivalent of 3 column volumes (20 ml) of buffer. The void volume was determined using Blue Dextran (Sigma), while the retention of >14kDa proteins was assessed using bovine heart cytochrome C (Sigma). Fractions were collected (100 µl) up to the retention volume for Cytochrome C and subsequently assayed for the presence of CD154 using a commercially available ELISA kit (Bender MedSystems, UK).

### NFkB (Rel A), cFos/cJun and STAT 3 activation in cholangiocytes following co incubation with sCD154/C4BP

Our previously published studies have shown that cholangiocyte stimulation via CD40 [3]–[5] results in transient NFkB (Rel A) activation whereas AP-1 (cJun/cFos) and STAT 3 activation is upregulated and sustained. In view of these findings, and the anti-apoptotic effect of C4BP we investigated NFkB (Rel A) cFos/cJun and STAT 3 activation following C4BP/sCD154 co-stimulation. Western blotting with specific antibodies to the functional transcription factors (see below) was carried out on cellular nuclear protein extracts. This approach has been previously described and shown to agree with results obtained by Electrophoretic Mobility Gel Shift Assay [4], [5]. Cholangiocyte monolayers were treated with sCD154 (final concentration 1 µg/ml), C4BP (final concentration 5 µg/ml) or both for either 4 or 24 hours. After these times, monolayers were harvested by scraping into cold PBS. Nuclear protein extracts were prepared as previously described [5] and protein content of each sample determined using the Micro Lowry Total Protein Kit (Sigma, Dorset, UK; according to the manufacturer's instructions). Samples (40 µg protein) were resolved on a 10% Bis-Acrylamide gel by SDS-PAGE, followed by transfer to nitrocellulose membrane (Amersham Pharmacia Biotech, Buckinghamshire, UK). Membranes were blocked overnight at 4°C in phosphate-buffered saline (PBS) containing 5% w/v non-fat dried milk and then washed (3×30 mins.) with 0.1%Tween-20/PBS, before incubating with primary antibodies. All incubations were for 1 hour at room temperature in Tween/PBS containing 5% w/v non-fat dried milk. The following antibodies were used; a) NFkB mouse monoclonal primary antibody (mAb) at 1/3000 dilution (Santa Cruz Biotechnology Inc. Cat. No. SC-8008). b) c-Fos rabbit polyclonal primary antibody at 1/2000 dilution (Santa Cruz Biotechnology Inc. Cat. No. SC-52). c) c-Jun rabbit polyclonal primary antibody at 1/2000 dilution (Santa Cruz Biotechnology Inc. Cat. No. SC-44). d) STAT3 mAb at 1/1000 dilution (Santa Cruz Biotechnology Inc. Cat. No. SC-8019). e) phospho-STAT3 mAb at 1/1000 dilution (Santa Cruz Biotechnology Inc. Cat. No. SC-8059). f) β-actin mAb at 1/2500 dilution (Sigma, Cat. No. A5441).

Following incubation with primary antibodies, the membranes were washed as described above and mAb detected using horse radish peroxidase (HRP) conjugated rabbit anti mouse IgG (1/1000 for 1 hour at room temperature). Primary polyclonal rabbit antibodies were detected using HRP conjugated goat anti rabbit IgG (1/1000 dilution for 1 hour at room temperature; Dako Ltd, Cambridge, UK). Membranes were then washed as before and protein bands visualised using an enhanced chemiluminescence detection system (Pierce Perbio, UK) followed by exposure to Hyperfilm-ECL (Amersham Pharmacia Biotech). Quantification of the protein bands was determined using densitometry scanning (BioRad 2000 Gel Doc system). Equality of protein loading onto membranes and complete transfer was confirmed by probing for β-actin and staining gels with Coomassie blue. All Western immunoblots were performed using nuclear extracts (NFkB, c-Fos and c-Jun) or cytoplasmic extracts (STAT3 and phospho-STAT3) prepared from cholangiocytes isolated from a minimum of three different human liver preparations.

### Immunolocalisation of C4BP and CD40 in human liver tissue

Immunohistochemistry was carried out according to a standard protocol on serial 4 micron sections of snap frozen acetone fixed liver tissue from normal (donor) liver or samples from patients with cholangiocarcinoma, hepatic metastases, primary sclerosing cholangitis or end stage alcoholic cirrhosis (n = 3–5 for each). Sections were first blocked (20 mins room temp.) with 20% normal rabbit serum (NRS) in Tris buffered saline pH 7.4 (TBS). Primary antibodies used were either (monoclonal) mouse anti human CD40 at 1∶50 dilution (Alexis Corp.) or mouse anti human C4BP at 1∶25 dilution (kindly provided by A.M.Blom). Incubation was continued for 1 hour. Following washing 3× with TBS, Alkaline phosphatase conjugated Rabbit anti mouse IgG (1∶25 diluted in TBS containing NRS) was added for 1 hour followed by mouse APAAP (1∶25). After 1 hour sections were washed in TBS pH 8.2, developed with Fast Red substrate, counterstained with Mayer's haematoxylin and mounted prior to assessment. Slides were coded and assessed blindly by a pathologist (SGH). Distribution of staining was recorded and the level of intensity of staining recorded using an established validated method of scoring where –ve relates to complete absence of staining and +++ represents the strongest observable level of expression.

In selected cases C4BP and CD40 were co localised using dual immunofluorescence. For these experiments a polyclonal goat anti human C4BP (R&D Systems) which gave staining patterns consistent with the mouse monoclonal reagent was substituted to avoid cross reactivity with conjugated secondary antibodies. Briefly snap frozen acetone fixed sections were blocked and then incubated with the goat anti human C4BP (1∶50 dilution) and mouse anti human CD40. Binding of primary antibodies was detected by addition of FITC conjugated donkey anti goat IgG and and PE conjugated rabbit anti mouse IgG (Dako). Controls included sections were primary antibodies were omitted or substituted for non immune serum or control immunoglobulin. Following staining sections were mounted inimmunofluorescent mountant (Dako) allowed to dry and examined using fluorescence microscopy.

### Statistical analysis

Statistical analysis was carried out using an unpaired t test. All primary cell culture experiments were carried out between 3 and 15 times as stated in text and figure legends.
